# Identification of novel antimicrobial resistance genes from microbiota on retail spinach

**DOI:** 10.1186/1471-2180-13-272

**Published:** 2013-12-01

**Authors:** Hillary F Berman, Lee W Riley

**Affiliations:** 1 Division of Infectious Disease and Vaccinology, School of Public Health, University of California, Berkeley, CA 94720, USA

**Keywords:** Antibiotic resistance, Gram negative bacteria, Metagenomic library

## Abstract

**Background:**

Drug resistance genes and their mobile genetic elements are frequently identified from environmental saprophytic organisms. It is widely accepted that the use of antibiotics in animal husbandry selects for drug resistant microorganisms, which are then spread from the farm environment to humans through the consumption of contaminated food products. We wished to identify novel drug resistance genes from microbial communities on retail food products. Here, we chose to study the microbial communities on retail spinach because it is commonly eaten raw and has previously been associated with outbreaks of bacterial infections.

**Results:**

We created metagenomic plasmid libraries from microbiota isolated from retail spinach samples. We identified five unique plasmids that increased resistance to antimicrobial drugs in the *E. coli* host. These plasmids were identified in *E. coli* that grew on plates that contained ampicillin (pAMP), aztreonam (pAZT), ciprofloxacin (pCIP), trimethoprim (pTRM), and trimethoprim-sulfamethoxazole (pSXT). We identified open reading frames with similarity to known classes of drug resistance genes in the DNA inserts of all 5 plasmids. These drug resistance genes conferred resistance to fluoroquinolones, cephalosporins, and trimethoprim, which are classes of antimicrobial drugs frequently used to treat human Gram negative bacterial infections. These results show that novel drug resistance genes are found in microbiota on retail produce items.

**Conclusions:**

Here we show that microbiota of retail spinach contains DNA sequences previously unidentified as conferring antibiotic resistance. Many of these novel sequences show similarity to genes found in species of bacteria, which have previously been identified as commensal or saprophytic bacteria found on plants. We showed that these resistance genes are capable of conferring clinically relevant levels of resistance to antimicrobial agents. Food saprophytes may serve as an important reservoir for new drug-resistance determinants in human pathogens.

## Background

The spread of antimicrobial resistance genes has made previously manageable bacterial infections increasingly more difficult to treat. In addition, there has been a gradual decline in the development of new antimicrobial drugs, especially against Gram negative bacterial pathogens. The identification of genes in Gram negative bacteria that confer resistance to cephalosporins, carbapenems, and fluoroquinolones has created fears that we are returning to the pre-antibiotic era [1]. These multidrug-resistant infections often occur in hospitals and are frequently caused by species belonging to the normal microbiota of the human host [1,2]. This suggests that the microbiota of the patients themselves is the reservoir for many of the organisms that cause hospital acquired infections. Furthermore, recent work has demonstrated that the intestinal microbiota of humans and food animals are a reservoir of drug resistance genes [3]. Consequently, a better understanding of how drug resistance genes enter the human microbiota is imperative to better prevent drug resistant infections.

Drug resistance genes and their mobile genetic elements are frequently identified from environmental saprophytic organisms. These include samples taken from soil, water, and wild animals [4-6]. Additionally, these genes have been identified in environmental samples from ancient and pristine environments – samples that have never been exposed to human activity [4,5,7]. Due to the great diversity of antibiotic resistance genes found in environment, it has been hypothesized that environmental microbes serve as a reservoir of drug resistance genes and that a few then enter human pathogens [4,5]. These drug resistance genes are spread between bacteria via mobile genetic elements, such as plasmids, transposons, and integrons [4-6,8]. The detection of mobile genetic elements and drug resistance genes in the environment has led to numerous studies and policies to address the effects of environmental exposure to antimicrobial agents on human pathogens [9,10].

It is widely accepted that the use of antibiotics in animal husbandry selects for drug resistant microorganisms, which are then spread from the farm environment to humans through the consumption of contaminated food products [11]. Numerous studies of bacterial pathogens in food products, such as *Campylobacter* and *Salmonella*, have demonstrated that the use of antimicrobial drugs in agriculture can result in drug resistant infections in humans [9-13]. However, the majority of studies have been limited to species of zoonotic pathogens that cause foodborne disease and these studies are frequently done as part of national surveillance programs for food safety. Species of bacteria that are not usually considered foodborne pathogens, but nonetheless are found in both the human and food product microbiota, are usually not included in studies of drug resistant bacteria in retail food products. Studies of microbiota of animals demonstrated that commensal organisms are a reservoir of antimicrobial drug resistance genes [3]. These studies include the identification of antimicrobial resistance genes from animal feces including chickens and cows [3,14-16]. Animal manure is frequently used as fertilizer in agriculture and may contribute to the spread of drug resistance genes. The spread of drug resistance genes by commensal bacteria on food products is an area that requires further study [11].

Produce items, which are frequently eaten raw, are one way consumers are exposed to microbiota on retail food products [17]. Previous work has shown that the normal microbiota of retail produce items harbors clinically relevant drug resistance genes [18]. However, previous studies have relied on PCR based methods to identify known drug resistance genes, which limits the number and types of drug resistance that could potentially be identified. Other studies used functional metagenomic libraries to identify novel antimicrobial resistance genes from environmental samples in a sequence independent manner [19,20]. We wanted to apply this sequence independent approach to investigate the presence of antimicrobial resistance genes on retail spinach. We chose to study the microbial communities on retail spinach because it is commonly eaten raw and has previously been associated with outbreaks of bacterial infections [21].

To do this, we made two metagenomic plasmid libraries with DNA isolated from the microbiota of retail spinach. One library was made from a cultured sample of spinach microbiota while the other was made in a culture independent manner. We then screened these libraries for their ability to confer resistance to antibiotics to an *E. coli* host.

## Results and discussion

### Isolation of antibiotic resistant clones

The first plasmid library, which was constructed from a cultured sample, contained 160 Mb of inserted DNA. The second library, which was constructed from an uncultured sample, contained 140 Mb of inserted DNA. We first constructed a cultured library because we wished to enrich for microbial DNA to increase the chances of cloning DNA sequences that contained drug-resistance genes. We constructed a library from an uncultured sample because we wanted to identify potential drug-resistance genes from bacterial organisms that cannot be cultivated in artificial medium. From the cultivated library, we isolated four different antimicrobial resistance-conferring clones. From the uncultivated library, we isolated one additional antimicrobial resistance-conferring clone.

The mean size of the plasmid DNA inserts in both libraries was two Kb. We identified five unique plasmids that conferred increased drug resistance (minimum inhibitory concentration or MIC) to the host *E. coli*. Each plasmid was named after the antimicrobial agent to which it conferred resistance (Table 1). These plasmids were identified in *E. coli* that grew on plates that contained ampicillin (pAMP), aztreonam (pAZT), ciprofloxacin (pCIP), trimethoprim (pTRM), and trimethoprim-sulfamethoxazole (pSXT). The plasmids pAMP, pAZT, pCIP, and pTRM were isolated from the library made from a cultured sample. The plasmid pSXT was isolated from the library made from an uncultured sample.

**Table 1:** **Minimum inhibitory concentrations (MIC) for *E. coli *** **containing the indicated plasmids**

**Drug resistant plasmid Name**	**Antimicrobial drug**	**Drug resistance plasmid MIC (ug/ml)**	**Empty vector MIC (ug/ml)**	**Fold increase**
pAMP	ampicillin	16	4	4
pAMP	piperacillin	3	2	1.5
pAMP	cefotaxime	<.25	<.25	0
pAMP	ceftazidime	<.5	<.5	0
pAMP	cefepime	0.064	0.094	0.68
pAMP	Aztreonam	0.19	0.125	1.5
pAMP	Imipenem	0.25	0.25	0
pCIP	ciprofloxacin	0.125	<0.002	>62.5
pCIP	levofloxacin	0.38	0.012	31.7
pCIP	Ampicillin	6	4	1.5
pCIP	piperacillin	3	2	1.5
pCIP	ceftazidime	<.5	<.5	0
pCIP	cefotaxime	<.25	<.25	0
pCIP	cefepime	0.125	0.094	1.3
pCIP	Imipenem	0.25	0.25	0
pTMP	trimethoprim	>32	0.124	>258
pTMP	trimethoprim- sulfamethoxazole	0.5	0.064	7.8
pAZT	Aztreonam	12	0.125	96
pAZT	cefepime	1	0.094	10.6
pAZT	ceftazidime	16	0.5	32
pAZT	piperacillin	12	2	6
pSXT	trimethoprim-sulfamethoxazole	1	0.064	15.6
pSXT	trimethoprim	>32	0.125	>256
pPRP:1B	ciprofloxacin	0.125	0.016	7.8
pPRP:1B	levofloxacin	0.25	0.047	5.3

MICs in the presence of the plasmid with the metagenomic DNA insert compared to the empty vector (pSMART or 1B) are shown. In accordance with manufactures recommendations, less than a 4-fold increase was considered to be within the margin of error.

pAMP increased the MIC of ampicillin 4 fold (4 ug/ml to 16 ug/ml). pAZT increased the MIC of the host strain 96 fold to aztreonam (.125 ug/ml to 12 ug/ml), 10 fold to cefepime (.096 ug/ml to 1 ug/ml), and 6 fold to piperacillin (2 ug/ml to 12 ug/ml). Additionally, pAZT encoded an ESBL phenotype as measured by the ceftazidime, ceftazidime/clavulanic acid ESBL Etest, (TZ 16 ug/ml and TZL 1 ug/ml). pCIP caused a 62-fold increase in resistance to ciprofloxacin (<.002 ug/ml to .125 ug/ml) as well as a 31-fold increase in resistance to levofloxacin (.012 ug/ml to .38 ug/ml). The MIC of trimethoprim for *E. coli* carrying pTRM increased >258 fold (.124 ug/ml to >32 ug/ml) and 7 fold to trimethoprim-sulfamethoxazole (.064 ug/ml to .5 ug/ml). pSXT caused a 15-fold increase in resistance to trimethoprim-sulfamethoxazole (.064 ug/ml to 1 ug/ml) as well as an >256-fold increase in resistance to trimethoprim alone (.125 ug/ml to >32 ug/ml).

### Identification of antibiotic resistance genes and phylogenetic analysis

**Figure 1:**
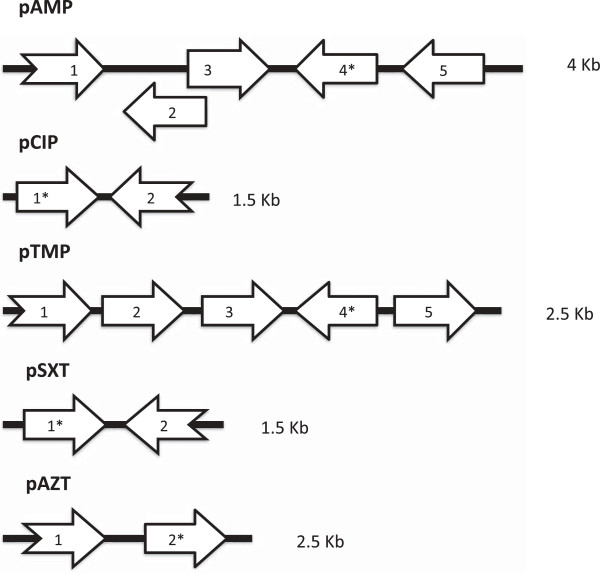
**Diagram of open reading frames and DNA insert size.** Open reading frame labels correspond to those in Table 2. Broken arrows correspond to truncated open reading frames. Open reading frames marked with a * show similarity to known drug resistance genes. Figure not drawn to scale.

We identified open reading frames with similarity to known classes of drug resistance genes in DNA inserts of all 5 plasmids. They are summarized in Figure 1 and Table 2.

**Table 2:** Open reading frames identified in each of the 5 drug resistance-conferring plasmids

**Plasmid**	**ORF**	**Predicted protein length**	**Most similar protein, Genbank accession number**	**Organism**	**E-value**	**AA identity**
pAMP	1	95	Putative Iron-Chelator Esterase EL859857.1	*Bacillus Subtilis subsp inaquoaorm*	7 e-55	99%
pAMP	2	230	Putative Sodium Dependent Transporter ZP_2118733.1	*Bacillus Subtilis subsp inaquoaorm*	2 e-156	98%
pAMP	3	378	Sugar Efflux Transporter YP_001907636.1	*Erwinia tasmaniensis Et1/99*	0.00	89%
pAMP	4	293	Extended Sepctrum Beta-lactamase ERP-1 AAAL86999.1	*Erwinina perscina*	2 e-152	81%
pAMP	5	303	Peptidyl-Dipeptidase YP_001907684.1	*Erwinia tasmaniensis Et1/99*	8 e-154	86%
pCIP	1	216	Pentapeptide-Repeat Containing Protein YP_006790167.1	*Exiguobacterium antarticum*	9 e-139	86%
pCIP	2	212	Beta-lactamase Domain Containing Protein YP_006790168	*Exiguobacterium antarticum*	1 e-144	96%
pTMP	1	128	Diemethyladenoanine Transferase YP_001906668.1	*Erwinia tasmanienaia*	1 e-82	94%
pTMP	2	125	Protein ApaG (Protein Cor D) YP_003532282.1	*Erwinia amylovora CFBP1430*	2 e- 78	88%
pTMP	3	234	Bis (5′-nucleoayl) Tetraphosphotase YP_003740080.1	*Erwinia billingiae*	4 e- 151	88%
pTMP	4	171	Protein FolA YP_ 005801502.1	*Erwinia pyrifoliae DSM 12163*	3 e-115	93%
pTMP	5	91	Rhtb Family Transporter YP_003532285.1	*Erwinina amylovora CFB1430*	9 e-54	97%
pAZT	1	151	Hypothetical Protein BsI_26310 ZP_21117554.1	*Bacillus Subtilis subsp inaquoaorm*	2 e-108	99%
pAZT	2	651	Penicillin Binding Protein 2c ZP_21117553.1	*Bacillus Subtilis*	0.00	99%
pSXT	1	170	Dihydrofolate Reductase YP_002875292.1	*Pseudomonas fluorsencens*	4 e-112	94%
pSXT	2	139	Sodium: Dicarboxylate Symporter ZP_0778142.1	*Pseudomonas fluorsencens*	3 e-93	96%

OrFinder, BlastX and BlastP were used to identify each open reading frame.

In pAZT we identified a sequence with 94% identity at the nucleotide level to a gene that encodes penicillin-binding protein 1A identified in *Bacillus subtilis subsp. Spizizenii* [GenBank, gb|CP002905.1|]. The expression of altered penicillin binding proteins are known to confer resistance to beta-lactam and cephalosporin antibiotics in various clinically important pathogens [22]. However, the ability of this sequence to confer clinically relevant levels of cephalosporin resistance or an ESBL phenotype has not been previously reported.

In pAMP we identified a beta-lactamase gene with 71% identity to the ERP-1 gene that encodes a class A extended spectrum beta-lactamase found in *Erwinia persicin* [GenBank, gb|AY077733.1|] [23]. When transformed into an *E. coli* host, ERP-1 was reported to increase resistance to penicillins and cephalosporins, including piperacillin, cefotaxime, and ceftazidime [23]. Surprisingly, we found that pAMP did not increase the MIC of the host *E. coli* to piperacillin, ceftazidime, or cefotaxime. Also, pAMP did not increase the MIC of this host strain to any tested cephalosporin, monobactam, or carbapenem (cefepime, aztreonam, or imipenem). However, this isolate tested positive for the presence of a beta-lactamase by the nitrocefin assay. These results suggest that the novel sequence we identified in pAMP is distinct from ERP-1 in terms of the spectrum of drug-resistance phenotype it encodes. However, it is also possible that these observations are artifacts due to poor expression of the gene in a heterologous host.

Surprisingly, when the pCIP DNA sequence was submitted to BlastN, only two other sequences in the NCBI non-redundant nucleotide database were identified. The sequences were part of whole genome sequences of *Exiguobacterium antarcticum* and *Exiguobacterium sibiricum*. These species were identified in a frozen Antarctic lake and a core sample of the Siberian tundra [24,25]. When submitted to BlastP, a fluoroquinolone resistance protein from *Oceanobacillus sp. Ndiop* was identified. This quinolone resistance protein was a predicted pentapeptide repeat protein (PRP) [GenBank, ref|ZP_10910075.1|]. One known class of plasmid-mediated quinolone resistance conferring sequences is called QNR[26]. QNRs are pentapeptide repeat proteins and have been associated with extended spectrum beta-lactamases [26]. In addition to the PRP, the pCIP DNA insert contained a second open reading frame that showed similarity to a beta-lactamase domain containing protein (Figure 1). However, pCIP did not increase the MIC of the host *E. coli* to any of the tested beta-lactam antibiotics (ampicillin and piperacillin), cephalosporin antibiotics (ceftazidime, cefotaxime, cefepime), or carbapenem antibiotics (imipenem). Additionally, pCIP did not test positive for beta-lactamase production by the nitrocefin assay. This indicated that the predicted beta-lactamase is either not expressed or does not function as a beta-lactamase.

We subcloned the PRP sequence in pCIP (pPRP:1B) in order to confirm that the predicted PRP was able to confer resistance to ciprofloxacin. As compared to the empty vector, the pPRP:1B increased the MIC of ciprofloxacin of the host *E. coli* 7 fold (.016 to .125) and the MIC of levofloxacin 5 fold (.047 to .25 ug/ml). The MIC conferred by pPRP:1B to the host *E. coli* is consistent with previously reported MICs from other QNR sequences found in human pathogens [26].

We found that the pTRM DNA insert has 84% identity to a region of the *Erwinia pyrifoliae DSM 12163* complete genome [GenBank, emb|FN392235.1]. This region contains the *folA* gene, which encodes a dihydrofolate reductase (DHFR). DHFR is the target of trimethoprim [27]. The expression of a DHFR that is not susceptible to trimethoprim is a well-known mechanism of resistance [27]. However, the acquisition of the *folA* gene from *Erwinia pyrifoliae DSM 12163* has not previously been shown to confer resistance to trimethoprim.

Similarly, the DNA insert from pSXT has 88% identity to a region of the *Pseudomonas fluorescens SBW25* complete genome [28]. This region also encodes a predicted dihydrofolate reductase. This sequence from *Pseudomonas fluorescens SBW25* has not previously been shown to confer resistance to trimethoprim or trimethoprim sulfamethoxazole.

## Conclusions

Here we show that microbiota of retail spinach contains previously unidentified antibiotic resistance-conferring genes and that functional metagenomic libraries can be used to screen retail food products for drug resistance genes in a sequence independent manner. Furthermore, due to the limited amount of DNA that can be cloned into a plasmid library and the requirement that the drug resistance gene be expressed in a heterologous host, it is likely we only identified a fraction of drug resistance conferring genes present in our spinach samples.

Although none was identical in DNA sequence, many of these novel sequences show sequence similarity to genes found in species of bacteria that have previously been identified as commensal or saprophytic bacteria found on plants [23,28]. This suggests the sequences we identified are not the result of contamination from animals or humans. We showed that these resistance genes are capable of conferring clinically relevant levels of resistance to commonly used classes of antimicrobial agents, including cephalosporins and fluoroquinolones.

The novel antimicrobial resistance genes we identified include beta-lactamases, a pentapeptide repeat protein, a penicillin binding protein, and putative dihydrofolate reductase genes. These types of resistance mechanisms are some of the most common and clinically problematic mechanisms of drug resistance found in pathogens [27]. We do not know at this time if these genes will become clinically important, and one limitation of this study is that we did not analyze these genes for their potential for horizontal transfer to human pathogens. Recent functional genomics analysis of environmental soil samples has not only identified drug-resistance genes with identical nucleotide sequences from human pathogens, but also mobile gene sequences providing evidence for possible horizontal gene transfers [29]. Further studies using sequence independent methods to identify antimicrobial resistance genes from retail food products should be done to better understand the role of saprophytes as a reservoir for new drug-resistance genes.

## Methods

### Metagenome plasmid library construction and screening

Two metagenomic plasmid libraries of spinach microbiota were constructed. One was based on DNA extracted from cultured bacteria and the other was based on DNA extracted from uncultured spinach wash. The metagenomic DNA used to create the first library was obtained by washing twenty five grams of bagged “baby spinach” in PBS. A description of the spinach used to create the library has been previously published [18]. Briefly, the spinach was purchased from a supermarket located in Berkeley, California in 2007. They included organic as well as non-organic spinach. One milliliter of the PBS wash was then used to inoculate 50 ml TSB. This culture was grown at 37°C with shaking overnight. The culture was then centrifuged at 10,000 x g for 10 minutes. DNA was extracted from the resulting pellet by the phenol chloroform method.

The metagenomic DNA used to create the second library was obtained by washing six bags of “baby spinach” in two liters of PBS. Six different brands of spinach were purchased from three retailers located in Berkeley, California in 2011. The spinach was incubated in PBS at room temperature for two hours. The resulting wash was then filtered through sterilized cheesecloth and a sterilized coffee filter to remove spinach debris. The filtered wash was then centrifuged at 10,000 x g for 20 minutes. DNA was extracted directly from the resulting pellet with the Gnome DNA isolation Kit, MP Biomedical.

Two plasmid libraries with metagenomic DNA inserts were constructed in pSMART-LC kan vector in the *E. coli* host, E. Cloni (Lucigen corp., Middleton WI). The pSMART vector confers resistance to kanamycin and has transcriptional terminators flanking the cloning sites. Consequently, transcription of the cloned sequences requires a native promoter.

*E. coli* clones containing the two plasmid libraries were then screened for resistance to antimicrobial agents on Mueller Hinton agar plates containing one of the following 16 antimicrobial agents: ampicillin, carbenicillin, ticarcillin, amoxicillin/clavulanic acid, ticarcillin/clavulanic acid, cefotaxime, ceftazidime, aztreonam, meropenem, gentamicin, nalidixic acid, ciprofloxacin, trimethoprim, trimethoprim-sulfamethoxazole, chloramphenicol, or tetracycline. The phenotype of resistance to antimicrobial agents was confirmed by retransforming the recombinant plasmid into E. Cloni. Growth on Muller Hinton agar containing kanamycin was used as a positive control for transformation. The acquisition of drug resistance from the transformation of the plasmid was demonstrated by growth on Mueller Hinton agar containing the corresponding antimicrobial agent. We used the empty vector, pSMART, in the *E. coli* host as a negative control for antibiotic stability.

### Antimicrobial susceptibility testing

The MIC of each *E. coli* clone was determined by Etest (Biomerieux, France) according to manufactures recommendations. All Etests were repeated in at least two independent experiments. ATCC 29522, ATCC 700603, and ATCC 35218 were used for control as recommended by the manufacturer. In accordance with manufactures recommendations, less than a fourfold difference in MIC was considered to be with in the expected margin of error for this test.

### Nitrocefin test

A colony was spotted onto a sterile Petri dish and then covered with nitrocefin, as previously described [30]. E. Cloni containing the empty vector (pSMART) was used as a negative control.

*Sequencing and data analysis*: The sequence of the DNA insert in the resistance conferring plasmid was determined by primer walking at the University of California, Berkeley sequencing facility. The sequences were assembled with Geneious Version 5.6, (Biomatters, New Zealand). The insert sequences were then submitted to ORFinder and the BLAST suit of programs at NCBI [31]. The nucleotide sequences of the insert from each plasmid have been deposited in Genbank with the following accession numbers: pAMP: KF791056, pAZT: KF791057, pCIP: KF791058, pTRM: KF791059, pSXT: KF791060.

### Cloning

Standard protocols for ligation independent cloning in to vector 1B, QB3 Macrolab, University of California, Berkeley were used.

## Competing interests

The authors declare that they have no competing interests.

## Authors’ contributions

HB carried out the laboratory and bioinformatics studies and drafted the manuscript. LWR conceived of the study, and participated in its design and coordination and helped to draft the manuscript. Both authors read and approved the final manuscript.
